# Spatial heterogeneity can undermine the effectiveness of country-wide test and treat policy for malaria: a case study from Burkina Faso

**DOI:** 10.1186/s12936-016-1565-2

**Published:** 2016-10-19

**Authors:** Denis Valle, Justin Millar, Punam Amratia

**Affiliations:** School of Forest Resources and Conservation, University of Florida, 136 Newins-Ziegler Hall, Gainesville, FL 32611 USA

**Keywords:** Malaria diagnostics, Presumptive treatment, Test and treat, RDT, Microscopy, Spatial heterogeneity

## Abstract

**Background:**

Considerable debate has arisen regarding the appropriateness of the test and treat malaria policy broadly recommended by the World Health Organization. While presumptive treatment has important drawbacks, the effectiveness of the test and treat policy can vary considerably across regions, depending on several factors such as baseline malaria prevalence and rapid diagnostic test (RDT) performance.

**Methods:**

To compare presumptive treatment with test and treat, generalized linear mixed effects models were fitted to data from 6510 children under five years of age from Burkina Faso’s 2010 Demographic and Health Survey.

**Results:**

The statistical model results revealed substantial regional variation in baseline malaria prevalence (i.e., pre-test prevalence) and RDT performance. As a result, a child with a positive RDT result in one region can have the same malaria infection probability as a demographically similar child with a negative RDT result in another region. These findings indicate that a test and treat policy might be reasonable in some settings, but may be undermined in others due to the high proportion of false negatives.

**Conclusions:**

High spatial variability can substantially reduce the effectiveness of a national level test and treat malaria policy. In these cases, region-specific guidelines for malaria diagnosis and treatment may need to be formulated. Based on the statistical model results, proof-of-concept, web-based tools were created that can aid in the development of these region-specific guidelines and may improve current malaria-related policy in Burkina Faso.

**Electronic supplementary material:**

The online version of this article (doi:10.1186/s12936-016-1565-2) contains supplementary material, which is available to authorized users.

## Background

Presumptive treatment for malaria has historically been the norm throughout much of sub-Saharan Africa (SSA). However, multiple problems plague presumptive treatment of malaria. First, considerable overlap in symptoms exists between malaria and other diseases [e.g., pneumonia; 1]. As a result, people that require treatment for other diseases might be inadvertently treated for malaria and sent home, with important consequences in terms of morbidity (and potentially mortality) and cost to the individual [2–4]. Second, there is substantial concern that presumptive treatment can promote more rapid emergence of anti-malarial drug resistance [5, 6]. Finally, as SSA countries transition to using artemisinin-based combination therapy (ACT) as their primary drugs for malaria treatment, presumptive treatment might be financially unsustainable given the higher costs of ACT [4, 7].

As a result of these concerns, the World Health Organization (WHO) has strongly promoted test and treat [8] as the primary malaria treatment policy for SSA countries. In this policy, “every suspected malaria case should be tested” and “every confirmed case should be treated with a quality-assured anti-malarial medicine” [8]. Because microscopy (the gold standard method in the majority of SSA countries [9]) is often unavailable in remote rural settings, the implementation of test and treat has relied heavily on rapid diagnostic tests (RDTs) for parasitological diagnostics [4, 5]. Recent studies have shown that the use of RDTs can lead to substantially improved targeting of anti-malarials when compared to only clinical diagnosis [10, 11]. In addition, despite the increased costs associated with using RDTs, test and treat might still be a cost-effective approach if it reduces the waste associated with providing ACT to uninfected individuals and if it decreases patient costs associated with repeated visits to health facilities ([2] but see [12]).

Despite the benefits associated with using RDTs, it has been repeatedly documented that providers and patients often ignore negative RDT results because of the potentially high stakes associated with a false negative result [13–15]. Indeed, while presumptive treatment certainly leads to over-treatment, test and treat can lead to both under and over-treatment because of false negative and false-positives RDT results, respectively. A recent Cochrane review indicates that RDTs have good overall performance (i.e., sensitivity greater than 90 % and specificity greater than 95 % on average) but finds considerable variation between studies [16]. In particular, RDT performance varied substantially due to differences in the study population (e.g., treatment-seeking individuals versus a random sample of the population), the reference standard [e.g., microscopy or polymerase chain reaction (PCR)], type of RDT and RDT manufacturer, and environmental conditions (e.g., extreme temperature or humidity may damage RDT lots) [4, 9, 17]. As an example of potential limitations of RDTs, the widely used HRP2-based RDTs (e.g., Paracheck®) are *Plasmodium falciparum* specific and thus fail to detect other *Plasmodium* species, will fail to detect *P. falciparum* if parasites have a mutation or deletion of the HRP-2 gene, and may detect the HRP-2 protein long after parasitaemia has been cleared from the host [18].

The relative merits of test and treat compared to presumptive treatment will depend on multiple factors, including performance characteristics of the diagnostic tests (sensitivity and specificity), baseline infection prevalence, and costs (both direct and indirect) associated with false-positives and false-negatives. In this article, Demographic and Health Survey (DHS) data from Burkina Faso were used to show that there is substantial spatial heterogeneity, even within regions of the same country, in relation to baseline infection prevalence and diagnostic test performance. As a result, a countrywide test and treat policy may yield unacceptably high levels of false-negative results in some regions, suggesting that presumptive treatment might be a better alternative in these settings. Proof-of-concept, web-based tools were developed that can aid policy makers in developing region-specific malaria diagnostic and treatment policies.

## Methods

### Data

This analysis was based on DHS data collected in Burkina Faso in 2010 (available at [19]). These data were collected through a two-stage sampling design where the first stage consisted of sampling clusters (total of 574 *zones de dénombrement*), with probability proportional to population size, and the second stage involved sampling households within each cluster with equal probability based on a complete listing of all the households. Children between 6 and 59 months old were tested for malaria using microscopy and a *P. falciparum* HRP2 protein-based RDT (Paracheck®) after obtaining consent from the caregiver. RDT results were available 15 min after blood collection while microscopy slides were later evaluated at the *Centre National de Recherche et de Formation sur le Paludisme* (CNRFP), the reference laboratory for malaria in Burkina Faso. Two independent technicians read each blood slide and, in the case of discrepancy, a third microscopist evaluated the slide [20, 21]. In total, microscopy and RDT results were collected from 6510 children. Additional details regarding data collection and diagnostic tests can be found in [20, 21].

### Statistical models

Two malaria biomarkers [microscopy (M) and RDT (R)] were modelled as a function of individual level covariates (X) using probit mixed regression models, where the intercept and slope parameters were allowed to vary for each region. Only relatively simple covariates were included in the statistical models, such as fever in the previous two weeks (no = 0, yes = 1), age (categorized into five age groups: 6–11, 12–23, 24–35, 36–47, 48–60 months), gender (0 = girl, 1 = boy), and urban (no = 0, yes = 1). These covariates were chosen because they have been shown elsewhere to be important malaria predictors and because they can be readily assessed with minimal training [22, 23].

The model for each biomarker had the same overall specification. Let a binary response variable (either microscopy or RDT result) for the i-th individual in the j-th region be denoted by $y_{ij}$. Assume that:

$$y_{ij} \sim Bernoulli\left( {\Phi \left( {\beta_{0j} + \beta_{1j} x_{ij1} + \beta_{2j} x_{ij2} + \cdots } \right)} \right)$$

where $\Phi$ is the standard normal cumulative distribution function, $x_{ij1} , \ldots ,x_{ijP}$ are covariates, and $\beta_{0j} , \ldots ,\beta_{Pj}$ are regression parameters. Standard priors and hyper-priors for these parameters were adopted, namely:

$$\beta_{pj} \sim N\left( {\alpha_{p} , \tau_{p}^{2} } \right)$$

$$\tau_{p} \sim Unif\left( {0,100} \right)$$

$$\varvec{\alpha}\sim N\left( {0,{\varvec{\Sigma}}} \right)$$

where ${\varvec{\Sigma}}$ is a diagonal matrix with diagonal elements $[100,1,1, \ldots ,1].$

Notice that $\alpha_{p}$ summarizes the effect of covariate p across all regions while $\tau_{p}^{2}$ measures the variability of the region-specific effects $\beta_{pj}$. Although these models are fairly standard and can be fitted in a frequentist or Bayesian framework [24], a Bayesian framework was chosen to better represent parameter uncertainty in outputs. Regression slope estimates for which the 95 % credible interval did not include zero were judged to be statistically significant.

These individual models were combined using Bayes theorem. Let (M|X) denote the model for microscopy results (M) given covariates (X). Furthermore, let (R|M,X) denote the model for the RDT results (R) given microscopy results (M) and covariates (X). Then, the probability of malaria infection (assuming microscopy is the gold standard) given RDT results (R) and covariate values (X) is given by:

$$\begin{aligned} &p\left( {M = 1|R,X} \right) \\ \nonumber &= \frac{{p\left( {R|M = 1,X} \right)p\left( {M = 1|X} \right)}}{{p\left( {R|M = 0,X} \right)p\left( {M = 0|X} \right) + p\left( {R|M = 1,X} \right)p\left( {M = 1|X} \right)}} \end{aligned}$$

The approach described above was chosen because of the interpretability of the results (e.g., the RDT models reveal how RDT sensitivity and specificity are influenced by covariates) and because it enables interactions to emerge naturally (e.g., the effect of age on RDT performance may vary as a function of infection status). Additional details regarding these statistical models are provided in Additional file 1. All models were fitted, and figures were created, using customized R code [25].

One potential concern with the model described above is that sample size might not be large enough within each strata to allow for reliable estimation of the different regression parameters. This was not a significant hindrance to this analysis given that all strata had at least 30 individuals. Finally, a ten-fold, cross-validation exercise revealed that the model described above had better out-of-sample predictive performance than two other more standard statistical models (Additional file 2).

### Online tools

In order to broaden the application of this analysis, two online tools were developed that enable policy makers and other potential users to interact with our statistical modeling results. These tools were created using ‘Shiny’ [26], a freely available package in R that enables the creation of interactive web applications without requiring modellers to know HTML, CSS, or JavaScript. These proof-of-concept tools were created to help bridge the gap between our statistical models and actionable policy decisions.

## Results

**Fig. 1:**
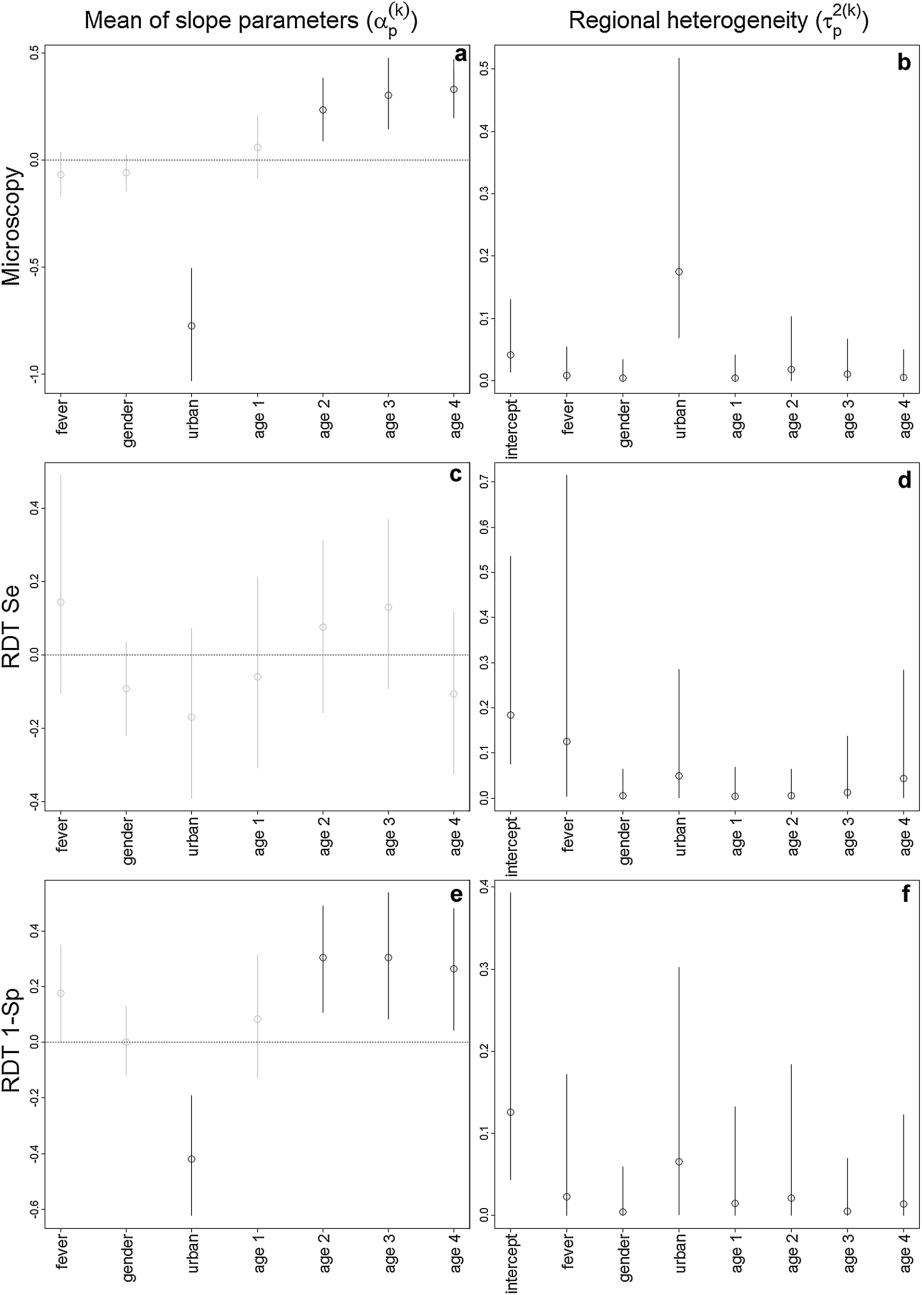
Parameter estimates (*circles*) and 95 % credible intervals (*vertical lines*). Results for the 3 different statistical models are displayed in each *row of panels*: microscopy (M|X; **a**, **b**), RDT sensitivity (R|M = 1,X; **c**, **d**), and one minus RDT specificity (R|M = 0,X; **e**, **f**). Age groups 1, 2, 3, and 4 refer to children 12–23, 24–35, 36–47, and 48–59 months old, respectively. *Left panels* depict the average of the random slope parameter p in model k ($\alpha_{p}^{\left( k \right)}$). Statistically significant parameters (i.e., 95 % credible intervals do not overlap with zero) are highlighted with *black lines* while non-significant results are depicted with *grey lines*. *Right panels* depict regional heterogeneity in effect sizes, represented by the variance of random parameter p in model k ($\tau_{p}^{2\left( k \right)}$). A detailed description of our statistical model is provided in Additional file 1. Se and Sp stand for sensitivity and specificity, respectively

Overall, individuals in urban areas tended to have a much lower risk of malaria and this risk tended to increase significantly with age (Fig. 1a). Furthermore, there was considerable heterogeneity between regions regarding malaria risk differences in rural and urban areas (Fig. 1b). In relation to RDT performance, the models indicate that the probability of a true positive RDT result (RDT sensitivity) was not consistently influenced by any of the covariates (Fig. 1c). On the other hand, the probability of a false positive RDT result was significantly higher for older children (two to four years old children) and was lower for individuals in urban areas (Fig. 1e). Both RDT sensitivity and specificity varied considerably from region to region (‘intercept’ in Fig. 1d, f).

**Fig. 2:**
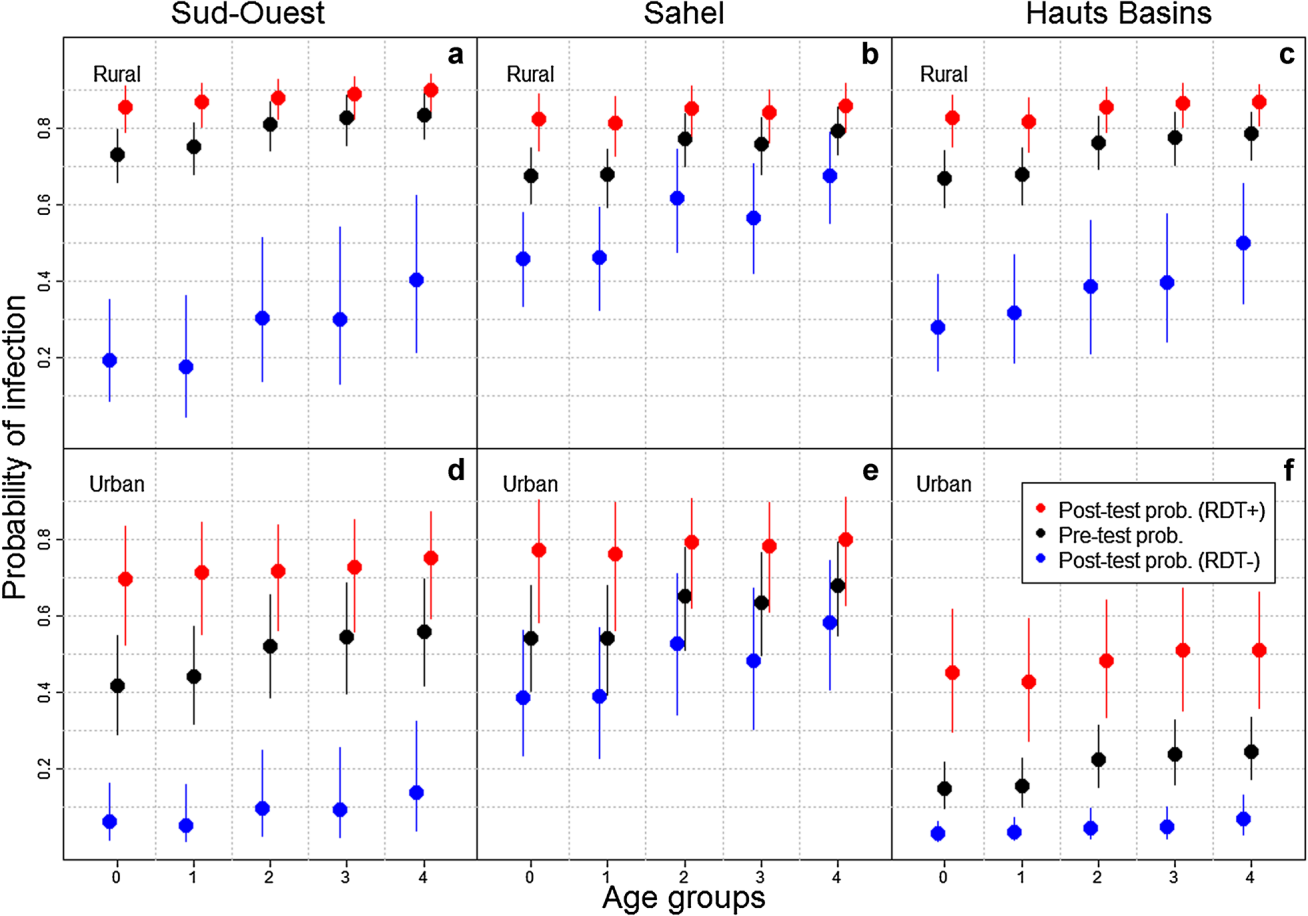
Probability of malaria infection for three regions in Burkina Faso (Sud-Ouest, Sahel, and Hauts Basins). Results are shown as a function of age group, urban/rural setting, and RDT result, for boys with no fever history in the previous 2 weeks. Age groups 0, 1, 2, 3, and 4 refer to children 6–11, 12–23, 24–35, 36–47, 48–59 months old, respectively. Pre-test probability of infection is shown in *black*, post-test probability of infection for RDT-negative individuals (RDT −) is shown in *blue*, and post-test probability of infection for RDT-positive individuals (RDT +) is shown in *red*. A large vertical distance between the *red* and *blue solid circles* indicates that RDT results are very informative regarding infection status

What do the findings described above imply in relation to the post-test probability of infection $p\left( {M |R,X} \right)$? RDT results substantially changed the probability of infection (red and blue circles in Fig. 2) compared to the pre-test probability of infection (i.e., baseline prevalence; black circles in Fig. 2), which indicate that RDTs are very informative regarding the likelihood of infection. However, individuals with a negative RDT result still have a 20–70 % chance of being infected in rural settings (blue solid circles in Fig. 2a–c). For instance, the infection probability of a four years old child with an RDT-negative result in rural areas of the Hauts Basins region (blue solid circles in Fig. 2c) can be equal to the infection probability of a RDT-positive child of the same age in the same region from an urban area (red solid circles in Fig. 2f). Three regions in Fig. 2 were selected to illustrate the patterns between infection status and the main covariates but an online tool was also developed to enable users to explore all factors included in our analysis for all regions (available through the website [27]).

In addition to variation between urban and rural areas within a particular region, this analysis also identified substantial differences between regions. For instance, RDT results seem more informative in the Sud-Ouest region than in the Sahel region (compare vertical distance between the blue and red solid circles in Fig. 2a, b, d, e). Similarly, although urban areas tend to have lower infection probability than rural areas, there seems to be substantial heterogeneity among urban areas. For example, probability of infection in urban areas of the Hauts Basins region is much lower than that in urban areas in Sud-Ouest and Sahel (compare Fig. 2d–f). These results suggest a complicated relationship between RDT outcomes and post-test probability of infection, indicating that a single malaria diagnosis and treatment policy even just for urban areas in Burkina Faso may not be effective. For instance, a test and treat policy might not be suitable for urban areas in the Sahel region, given the high probability of infection (>0.3) of RDT-negative children, whereas it might be a suitable option for urban areas in the Hauts Basin region.

The relevance of spatial heterogeneity for malaria diagnosis and treatment policy can be illustrated with a hypothetical example. For instance, if policy makers decided that it is unacceptable to use a diagnostic test with a probability of false-negative results above 30 % (for example), then presumptive treatment would be recommended for all rural areas in Burkina Faso and for urban settings in the Sahel and Est regions. In another online tool that we have developed (available through the website [28]), readers can explore the geographic implications regarding recommended presumptive treatment for different thresholds of the probability of false-negative results.

## Discussion

The statistical model for malaria microscopy confirms several of the relationships often described in malaria epidemiology in endemic regions, such as the increase in malaria prevalence with age [29] and generally higher prevalence in rural settings when compared to urban areas [30]. However, the impact of some of these factors (e.g., age and urban *vs* rural settings) on RDT performance is less commonly explored [but see 23, 31, 32]. The modelling results suggest that RDT specificity is higher for children in urban settings and younger children (Fig. 1e). This might be due to these children being less likely to have had a past malaria infection since false positives will typically arise on individuals with past malaria infections due to the relatively long time that the target antigen persists in the blood [33, 34]. Alternatively, these RDT results might be correct but microscopy might have failed to detect these infected individuals, which is likely to be a common phenomenon for individuals with low parasitaemia levels [35–37]. Finally, substantial geographical variation was observed regarding RDT sensitivity and specificity. Multiple reasons could explain this, including differences in the proportion of individuals with past exposure to malaria, exposure of RDTs to excessive heat and humidity during storage and distribution, intra-species diversity of the target antigen, and the parasite stage-specific expression of the antigen [4, 17, 38].

Most importantly, these multiple statistical models were combined to highlight the substantial spatial heterogeneity in malaria risk given RDT results. Under a countrywide test and treat policy, in some regions children with very similar infection probabilities might be denied treatment in a rural setting (due to a negative RDT result) but receive treatment in an urban setting (due to a positive RDT result). To avoid this, region-specific guidelines for malaria diagnosis and treatment might need to be developed, a process that can be potentially aided by the developed tool. Differently from past approaches that have relied on a threshold for the pre-test probability of clinical malaria [39], the proposed tool focuses on the probability of false-negative results to determine which areas a test and treat policy might be warranted. Obviously there are a number of other considerations that should be taken into account when determining policies for malaria diagnosis and treatment. For instance, the results of this study, in conjunction with direct and indirect cost information, would be very informative for policy makers if used in a decision-theoretic framework. In particular, local heterogeneities (e.g., longer travel times and lower wages in rural settings) may play an important role in determining what is most cost effective in each setting. Nevertheless, this analysis clearly illustrates important shortcomings of a uniform test and treat policy across Burkina Faso.

An important caveat to this study is that the data contain children that were sampled regardless of their symptoms and there was no information on symptoms on the day of the survey. Although presence of fever during the previous two weeks was controlled for (a variable that was surprisingly unimportant), baseline malaria prevalence and RDT performance are likely to be different for individuals that seek help in health facilities due to the presence of symptoms. Thus, using data on syndromic individuals would certainly be more appropriate when designing policies for malaria diagnosis and treatment at health facilities. Unfortunately, data over large geographical regions on treatment-seeking individuals that are tested with multiple diagnostic methods (i.e., microscopy and RDT) are scarce. Additional studies are warranted to determine if similar spatial heterogeneities are found when using syndromic data in Burkina Faso and in other malaria-endemic regions. Nevertheless, the results in this article are relevant for determining where a mass screen and treat strategy is likely to be more effective than a mass drug administration approach, a topic that has received renewed interest by policy makers and researchers and for which evidence is still relatively scarce in moderate to high malaria transmission areas ([40] but see [31, 41]). Finally, although this study focuses on children under five years, similar ideas are likely to be applicable for comparing intermittent screening and treatment (IST) as an alternative approach to diagnosis by symptoms only or intermittent preventive treatment for pregnant women [42–45].

One potential concern regarding this study refers to using microscopy as the gold standard method. Although routine microscopy can yield highly variable results, it is important to note that microscopy was conducted by the parasitology laboratory of the CNRFP, the reference laboratory for malaria in Burkina Faso. Staff at this centre participate in various quality control programmes in parasitology (e.g., proficiency testing programmes to comply with the College of American Pathologists and WHO-AFRO checklists) and are subject to rigorous internal and external quality control [21, 46]. Furthermore, DHS surveys across the majority of the SSA countries have relied on expert microscopy and these microscopy results have been extensively used by the scientific community as the gold standard (e.g., to create national malaria prevalence maps). Finally, expert microscopy is commonly used as the gold standard method against which RDT is evaluated [20, 34, 47, 48]. Although even expert microscopy can miss a large proportion of asymptomatic carriers [41], often because of low total parasite density [35–37], this does not explain the high proportion of positive microscopy but negative RDT results (i.e., false-negative results) found here in some regions.

Why is there such a high proportion of false-negative results? There may be multiple non-exclusive reasons. RDTs might have been exposed to extreme conditions of temperature and humidity which may have compromised its performance (as documented for Burkina Faso in [49]). The prevalence of other *Plasmodium* species might have been high (e.g., a 13.2 % prevalence of *Plasmodium malariae* was found in a village in rural Burkina Faso in 2010 [50]), undermining a RDT that exclusively targets *P. falciparum*. Finally, it has been shown that the performance of RDT (both pLDH-based and HRP-2-based) is reduced in patients with lower parasite density [31, 51, 52]. While patients with false negative results may be deemed clinically irrelevant given their likely low parasite density [51, 53], this reasoning does not apply here because this study focuses on children under five and even low parasite densities are likely to result in fever [51] and lower mean haemoglobin levels [54] for this age group. Furthermore, it has been shown that malaria transmission readily occurs at low parasitaemia [55, 56] and that low parasitaemia infections significantly contribute to malaria transmission due to the high prevalence of these infections [35, 56, 57]. These findings suggest that, even if false negative results arise from low parasitaemia, it remains important to treat these individuals. It is also important to realize that, for children with very high malaria prevalence (e.g., older children in rural areas), even a RDT with high sensitivity is likely to generate a relatively high probability of false negatives [58]. For instance, if sensitivity is 0.95 and malaria prevalence is 0.8, Bayes theorem indicates that the probability of false negative is

$$\begin{aligned} &p\left( {M = 1|R = 0} \right) \\ &\ge \frac{{p\left( {R = 0|M = 1} \right)p\left( {M = 1} \right)}}{{p\left( {R = 0|M = 1} \right)p\left( {M = 1} \right) + p\left( {R = 0|M = 0} \right)p\left( {M = 0} \right)}} \\ &= \frac{{\left( {1 - 0.95} \right) \times 0.8}}{{\left( {1 - 0.95} \right) \times 0.8 + 1 \times \left( {1 - 0.8} \right)}} = \frac{1}{6} \end{aligned}$$

In other words, among all RDT negative children, at least one out of six will actually be infected with malaria. In this calculation, RDT specificity $p\left( {R = 0|M = 0} \right)$ was assumed to be 100 % but the probability of false negative results can be even higher for lower values of RDT specificity. Indeed, the above scenario is very optimistic. For instance, using the same DHS data from Burkina Faso, Samadoulougou et al. [20] have shown that RDT sensitivity was actually closer to 90 % (89.9 %, with 95 % confidence interval CI of 89–90.8) and specificity was very low (50.4 %, 95 % CI of 48.3–52.6).

Another important remark is that the 2010 DHS data for Burkina Faso was predominantly collected during the rainy season. As a result, the well documented seasonal differences in malaria risk and RDT performance in Burkina Faso [32, 41, 44, 51–53, 56, 59–65] were not accounted for in the statistical models. These seasonal differences can be as dramatic as the geographical differences described here. For instance, similar to the observed pattern for the urban *vs* rural areas in the Hauts Basins region, a negative RDT test has been shown to reduce the probability of malaria to almost zero during the dry season but not in the rainy season [51]. An important implication of not taking into account seasonality in our models is that any policy derived from these tools is likely to be applicable only for the rainy season. Finally, DHS data were collected during approximately the same time period for each region except for the Centre, Centre-Nord, Nord, and Plateau Central regions. Because of these differences regarding when data were collected in each region, geographical comparisons involving the regions cited above should be interpreted carefully as they may be confounded with seasonality effects.

## Conclusion

Similar to the findings in [39, 51], the analysis presented here suggests that a generalized test-based policy should not be used uniformly across all contexts. In particular, even with improved diagnostic methods (e.g., positive control wells; [66]), the ‘back-of-the-envelope’ calculation above reveals how the probability of false-negative results will still be relatively large when prevalence is high. Unfortunately, because prevalence varies significantly even within the same region (e.g., according to age groups, season, rural *vs* urban), developing sound and straightforward diagnostic guidelines remains an important challenge. This article has shown how different statistical models can come together to inform context specific guidelines for malaria diagnosis and treatment, potentially improving the use of resources (e.g., reducing wasted RDTs in regions where false-negative probabilities are very high) and reducing malaria burden. Ultimately, bridging the gap between information users and these statistical models will be critical to foster evidence-based decision making and better resource allocation.

## Additional files

**Additional file 1.** Description of statistical models.

**Additional file 2.** Model validation.
