# Supplementary material for: Spatial heterogeneity can undermine the effectiveness of country-wide test and treat policy for malaria: a case study from Burkina Faso
Source: Malar J. 2016 Oct 19;15:513. doi: 10.1186/s12936-016-1565-2 (PMC5070201; doi:10.1186/s12936-016-1565-2)
Supplement: Supplementary file 1 — Additional file 1. Description of statistical models. [file 12936_2016_1565_MOESM1_ESM.docx]

**Additional File 1. Description of statistical models**

Our statistical model focused on two malaria biomarkers: Microscopy (M) and RDT (R). To jointly model these two outcomes, we adopt the following factorization for individual i in region j:

$$p\left( M_{ij},R_{ij}|\boldsymbol{x}_{\boldsymbol{ij}}\boldsymbol{,}\boldsymbol{\beta}_{\boldsymbol{j}}^{\left( \boldsymbol{M} \right)}\boldsymbol{,}\boldsymbol{\beta}_{\boldsymbol{j}}^{\left( \boldsymbol{R}_{\boldsymbol{0}} \right)}\boldsymbol{,}\boldsymbol{\beta}_{\boldsymbol{j}}^{\left( \boldsymbol{R}_{\boldsymbol{1}} \right)} \right)=p\left( R_{ij}|M_{ij},\boldsymbol{x}_{\boldsymbol{ij}}\boldsymbol{,}\boldsymbol{\beta}_{\boldsymbol{j}}^{\left( \boldsymbol{R}_{\boldsymbol{0}} \right)}\boldsymbol{,}\boldsymbol{\beta}_{\boldsymbol{j}}^{\left( \boldsymbol{R}_{\boldsymbol{1}} \right)} \right)\times p\left( M_{ij}|\boldsymbol{x}_{\boldsymbol{ij}}\boldsymbol{,}\boldsymbol{\beta}_{\boldsymbol{j}}^{\left( \boldsymbol{M} \right)} \right)$$

where $\boldsymbol{x}_{\boldsymbol{ij}}$ is a vector containing covariates, and $\boldsymbol{\beta}_{\boldsymbol{j}}^{\left( \boldsymbol{M} \right)}\boldsymbol{,}\boldsymbol{\beta}_{\boldsymbol{j}}^{\left( \boldsymbol{R}_{\boldsymbol{0}} \right)}\boldsymbol{,}\boldsymbol{\beta}_{\boldsymbol{j}}^{\left( \boldsymbol{R}_{\boldsymbol{1}} \right)}$ are vectors of regression parameters. We adopt the following probit model for the microscopy results $M_{ij}$:

$$M_{ij}\sim Bernoulli\left( \Phi\left( \boldsymbol{x}_{\boldsymbol{ij}}^{\boldsymbol{T}}\boldsymbol{\beta}_{\boldsymbol{j}}^{\left( \boldsymbol{M} \right)} \right) \right)$$

where $\Phi$ is the standard normal cumulative distribution function. In relation to RDT results $R_{ij}$, we created two probit models, one for $M_{ij}=0$ and the other for $M_{ij}=1$:

$$R_{ij}|M_{ij}=0\sim Bernoulli\left( \Phi\left( \boldsymbol{x}_{\boldsymbol{ij}}^{\boldsymbol{T}}\boldsymbol{\beta}_{\boldsymbol{j}}^{\left( \boldsymbol{R}_{\boldsymbol{0}} \right)} \right) \right)$$

$$R_{ij}|M_{ij}=1\sim Bernoulli\left( \Phi\left( \boldsymbol{x}_{\boldsymbol{ij}}^{\boldsymbol{T}}\boldsymbol{\beta}_{\boldsymbol{j}}^{\left( \boldsymbol{R}_{\boldsymbol{1}} \right)} \right) \right)$$

One way to interpret these models is that the probability of a false positive result from RDT (one minus specificity) is given by $\Phi\left( \boldsymbol{x}_{\boldsymbol{ij}}^{\boldsymbol{T}}\boldsymbol{\beta}_{\boldsymbol{j}}^{\left( \boldsymbol{R}_{\boldsymbol{0}} \right)} \right)$ while the probability of a true positive result from RDT (sensitivity) is given by $\Phi\left( \boldsymbol{x}_{\boldsymbol{ij}}^{\boldsymbol{T}}\boldsymbol{\beta}_{\boldsymbol{j}}^{\left( \boldsymbol{R}_{\boldsymbol{1}} \right)} \right)$. For all three models, we impose random effects priors for the intercept and slope parameters:

$$\boldsymbol{\beta}_{\boldsymbol{j}}^{\left( \boldsymbol{k} \right)}\sim N\left( \boldsymbol{\alpha}^{\left( \boldsymbol{k} \right)},\boldsymbol{\Omega}^{\left( \boldsymbol{k} \right)} \right)$$

where $\boldsymbol{\Omega}^{\left( \boldsymbol{k} \right)}$ is a diagonal matrix with diagonal elements $\left\{ \tau_{0}^{2\left( k \right)},\ldots,\tau_{P}^{2\left( k \right)} \right\}$ and $k=\left\{ M,R_{0},R_{1} \right\}$. Finally we specify our hyper-priors as:

$$\tau_{p}^{\left( k \right)}\sim Unif\left( 0,100 \right)$$

$$\boldsymbol{\alpha}^{\left( \boldsymbol{k} \right)}\sim N\left( \boldsymbol{0},\boldsymbol{\Sigma} \right)$$

where $\boldsymbol{\Sigma}$ is a $P\times P$ diagonal matrix with diagonal elements $\left\{ 100,1,\ldots,1 \right\}$. P is the number of regression parameters.

We created a customized Gibbs sampler in R [^1^](#_ENREF_1) to fit the models described above. For each model, a single chain was run for 10,000 iterations and the first 1,000 iterations were discarded as burn-in. Due to the large number of parameters in these models, convergence was assessed by visual examination of trace-plots of the summary parameters $\boldsymbol{\alpha}$ and $\boldsymbol{\Omega}$.

References

1. R Core Team, 2013. R: A language and environment for statistical computing.: R Foundation for Statistical Computing, Vienna, Austria.
