# Supplementary material for: Spatial heterogeneity can undermine the effectiveness of country-wide test and treat policy for malaria: a case study from Burkina Faso
Source: Malar J. 2016 Oct 19;15:513. doi: 10.1186/s12936-016-1565-2 (PMC5070201; doi:10.1186/s12936-016-1565-2)
Supplement: Supplementary file 2 — Additional file 2. Model validation. [file 12936_2016_1565_MOESM2_ESM.docx]

**Additional File 2. Model validation**

To validate our model, we performed a 10-fold cross validation exercise. In this exercise, we randomly removed 10% of the data and fitted the model to the remaining 90%. Then, using the estimated parameters, we calculated the log-likelihood for each left-out observation. This procedure was executed 10 times, each time leaving a distinct subset of the data out for validation purposes. The sum of the log-likelihood over the left-out sample is used here as a measure of overall out-of-sample predictive skill of the statistical model.

We compared our Bayesian model to two other more standard statistical models. The first one consisted in a simple logistic regression (fitted using the “glm” function in R) while the second model consisted of a logistic regression with region-specific random intercepts (fitted using the “glmer” function within the “lme4” package in R). Both of these logistic regression models used microscopy results as the response variable and the following covariates: RDT, fever in past 2 weeks, gender, urban vs. rural, and age (discretized into 5 age groups).

The out-of-sample sum of the log-likelihood was equal to -3008, -2970, and -2948, for the simple logistic regression, the logistic regression with random intercepts, and our Bayesian model, respectively. These results suggest that the Bayesian model has better overall performance than the other more standard models. The results disaggregated for each validation sample show that Bayesian model always outperformed the simple logistic regression and that it performed better than the logistic regression with random intercepts in 8 out of the 10 validation samples (Table A1). These results, together with the greater interpretability of the Bayesian model (e.g., enabling the distinction between factors that influence RDT specificity from those that influence malaria risk), support our use of the Bayesian model in the main manuscript.

Table A1. Log-likelihood for different validation samples for the Bayesian model, simple logistic regression model, and the mixed effects logistic regression model. The best method for each validation sample is highlighted in bold.

| Validation sample | Bayesian model | Simple Logistic | Logistic random intercepts |
| --- | --- | --- | --- |
| 1 | **-321** | -326 | -324 |
| 2 | **-285** | -297 | -288 |
| 3 | **-313** | -319 | -316 |
| 4 | **-272** | -274 | -273 |
| 5 | -282 | -283 | **-277** |
| 6 | **-272** | -278 | -276 |
| 7 | -309 | -312 | **-308** |
| 8 | **-299** | -303 | -301 |
| 9 | **-304** | -313 | -311 |
| 10 | **-290** | -303 | -297 |
| Total | **-2948** | -3008 | -2970 |
